# A Novel Small-Molecule Inhibitor Targeting CREB-CBP Complex Possesses Anti-Cancer Effects along with Cell Cycle Regulation, Autophagy Suppression and Endoplasmic Reticulum Stress

**DOI:** 10.1371/journal.pone.0122628

**Published:** 2015-04-21

**Authors:** Jong Woo Lee, Hee Sun Park, Sin-Aye Park, Seung-Hee Ryu, Wuyi Meng, Juliane M. Jürgensmeier, Jonathan M. Kurie, Waun Ki Hong, Julie L. Boyer, Roy S. Herbst, Ja Seok Koo

**Affiliations:** 1 Section of Medical Oncology, Department of Internal Medicine, Yale Comprehensive Cancer Center, Yale School of Medicine, Yale University, New Haven, CT 06520, United States of America; 2 Department of Radiation Oncology, University of Ulsan College of Medicine, Asan Medical Center, Seoul, Republic of Korea; 3 Department of Molecular Biophysics and Biochemistry, Yale University, New Haven, CT 06520, United States of America; 4 Department of Thoracic/Head and Neck Medical Oncology, The University of Texas MD Anderson Cancer Center, Houston, TX 77030, United States of America; 5 Translational Research Program, Yale Comprehensive Cancer Center, New Haven, CT 06520, United States of America; Baylor College of Medicine, UNITED STATES

## Abstract

Lung adenocarcinoma, the most common subtype of lung cancer, is the leading cause of cancer death worldwide. Despite attempts for the treatment of lung cancer which have been accumulating, promising new therapies are still needed. Here, we found that cyclic-AMP response element-binding protein (CREB)-CREB binding protein (CBP) transcription factors complex inhibitor, Naphthol AS-TR phosphate (NASTRp), is a potential therapeutic agent for lung cancer. We show that NASTRp inhibited oncogenic cell properties through cell cycle arrest with concomitant suppression of tumor-promoting autophagy with down-regulations of Atg5-12 and Atg7, and accumulation of p62 in human lung cancer cell lines. In addition, NASTRp induced expression of endoplasmic reticulum stress markers such as DDIT3/CHOP, and led to apoptosis along with Bim induction. These findings suggest that transcription factor/co-activator complex, CREB-CBP, can be a potential therapeutic target and its inhibition could be a novel therapeutic strategy for lung cancer.

## Introduction

Lung cancer is one of the leading causes of cancer mortality worldwide, and it is estimated that 159,480 lung cancer patients will die in the United States in 2013 [1]. Approximately 25% of lung adenocarcinomas, a dominant form of lung cancer harbor oncogenic *KRAS* mutations, and this poses a significant therapeutic challenge, as *KRAS* mutations are generally associated with poor prognosis and resistance to chemotherapy [2, 3]. Direct pharmacologic targeting of activated KRAS mutant protein has been unsuccessful so far, thus, alternative approaches to block KRAS activation signaling pathway are being considered. Notably, mutant KRAS drives activation of cyclic-AMP response element-binding (CREB) through RAF/MEK/ERK signaling pathway to force cancer cell growth and survival. Thus, one option to inhibit growth of KRAS mutant tumors may be to target transcription factors (e.g. CREB), which are often the final regulator of multiple signaling processes, and could potentially be targeted regardless of alterations of upstream signaling components involved in cancer development, progression and invasion/metastasis.

CREB is a critical transcription factor involved in normal homeostasis [4–6], metabolism [7], memory/learning [8], several cancers [9–12] and immune diseases [13]. Our previous studies showed that CREB is highly upregulated and hyperphosphorylated in most of the non-small cell lung cancer (NSCLC) tumor specimens and that this upregulation is significantly associated with poor survival rates [10–12]. CREB is phosphorylated at serine/threonine residues depending upon the stimuli from extracellular components and several upstream kinases. Activated/phosphorylated CREB recruits its transcription co-activator, CREB-binding protein (CBP) to a cAMP response element (CRE) region of target genes [14]. This recruitment of CBP is a critical step for the transcriptional activation of CREB [15]. Therefore, blocking the interaction between CREB-CBP could be an approach to inhibit CREB transcriptional activity. In fact, identification of small molecule inhibitors interfering with the formation of the CREB-CBP complex through targeting the KID and KIX domains of CREB and CBP, respectively, has been reported using an NMR screening approach [16]. In addition, we previously showed that one of these inhibitors, 2-Naphthol-AS-E phosphate (KG-501), which directly targets the KIX domain of CBP, resulted in a disrupted CREB-CBP complex, inhibited CREB-target gene induction, and inhibited IL-1β-mediated angiogenic activity in NSCLC [10].

With the aim of improving therapeutic attempts for lung cancer harboring KRAS mutant, we found a multi-functional transcription factor inhibitor named Naphthol AS-TR phosphate (NASTRp), targeting the CREB-CBP complex, as a potent anti-cancer agent for lung cancer. Collectively, NASTRp showed clear efficacy in multiple biological assays and could and will be a potential therapeutic approach for human cancers, especially for lung cancer.

## Materials and Methods

### Cell culture

Human lung cancer cell lines, A549, NCI-H1734, NCI-H1792, NCI-H441, NCI-H23, NCI-H1975 and NCI-H520 cells were obtained from the American Type Culture Collection (ATCC, Manassas, VA, USA). Normal human tracheobronchial epithelial (NHTBE) cells were obtained from the Lonza (Walkersville, MD, USA). Cell lines were passaged for less than 6 months following resuscitation and were not authenticated. All cancer cell lines were maintained under 5% CO_2_ at 37°C in RPMI-1640 medium (Life Technologies, Carlsbad, CA, USA) supplemented with 10% heat-inactivated fetal bovine serum (FBS, Sigma-Aldrich, St. Louis, MO, USA) and 1% Antibotic-Antimycotic (Anti-Anti, Life Technologies). NHTBE cells were cultured in BEBM supplemented with growth factors and hormones provided by manufactory (Lonza), and three-demensional organotypic air-liquid interface (ALI) cell culture method was utilized for NHTBE cell culture, as described previously [5, 17–19]. HEK293T cells were maintained in DMEM medium supplemented with 10% FBS and 1% Anti-Anti.

### Proliferation, colony formation and soft agar assays

Cells were seeded in 96 well plates at 2×10^3^ cells/well with RPMI-1640 medium supplemented with 5% heat-inactivated FBS and without Anti-Anti. Cells were treated with Naphthol AS-TR phosphate disodium salts (NASTRp, Sigma-Aldrich, N6125) as 0–80 μmol/L for 96 hours. Cell proliferation was measured with MTT or CellTiter Glo luminescent cell viability assay (Promega, Madison, WI, USA). Cell viability of vehicle-treated cells was set to 100% of proliferation. For colony formation assay, cells were seeded in 6 well plates at 1×10^3^ cells/well. Cells were treated with NASTRp as 0, 5, 10, and 20 μmol/L and were changed with fresh media containing NASTRp every other day for 10–17 days at which point 0.1% (wt/vol) crystal violet (Thermo Scientific, NJ, USA) was used to visualize colonies. Soft agar assay was performed as described previously [12]. Briefly, colonies were stained with p-iodonitrotetrazolium violet (Sigma-Aldrich) to select alive colonies and then, stained colonies were counted using Image J software (NIH, USA). A colony was defined as anything containing more than 10 cells, as indicated >50 pixels in Image J.

### Quantitative reverse transcription-PCR

qRT-PCR analysis was performed as described previously [11]. In brief, Total RNA was purified from cells using RNeasy Mini Kit (Qiagen, Valencia, CA, USA). Reverse transcription of total RNA was performed using the MMLV reverse transcriptase (Promega). Quantitative PCR was performed using SYBR Green PCR Core Reagents (Applied Biosystem, Life Technologies). All primers were purchased from Keck (New Haven, CT, USA). All reactions were performed in triplicate and ribosomal protein L32 cDNA levels were used as an endogenous control. The primer sequences used in qRT-PCR were as follows: Cyclin A2; forward; 5’-CCAAGAGGACCAGGAGAATA-3’, reverse; 5’-CGGTGACATGCTCATCATT-3’, Cyclin B1; forward; 5’-AGTCACCAGGAACTC GAAAA-3’, reverse; 5’-GTTACCAATGTCCCCAAGAG-3’, Cyclin D1; forward; 5’-CTTC AAATGTGTGCAGAAGG-3’, reverse; 5’-AGCGGTCCAGGTAGTTCAT-3’; Cyclin E2; forward; 5’-AGAGAGGAGGTCACCAAGAAA-3’, reverse; 5’-CAGGCAAAGGTGAAGGAT TA-3’, GRP78; forward; 5’-CACAGTGGTGCCTACCAAGA-3’, reverse; 5’- TGTCTTTTGTC AGGGGTCTTT-3’, CHOP; forward; 5’-AGCCAAAATCAGAGCTGGAA-3’, reverse; 5’-TGG ATCAGTCTGGAAAAGCA-3’, IRE1; forward; 5’-CTCTGTCCGTACCGCCC-3’, reverse; 5’- GAAGCGTCACTGTGCTGGT-3’, PERK; forward; 5’-TCATCCAGCCTTAGCAAACC-3’, reverse; 5’-ATGCTTTCACGGTCTTGGTC-3’, ATF6; forward; 5’-GCAGAAGGGGAGACAC ATTT-3’, reverse; 5’-TTGACATTTTTGGTCTTGTGG-3’, XBP1; forward; 5’-CGAATGAG TGAGCTGGAACA-3’, reverse; 5’-GGCCATGAGTTTTCTCTCGT-3’, L32; forward; 5’-CAC CAGTCAGACCGATATGT-3’, reverse; 5’-ACGTTGTGGACC AGGAACT-3’. The real-time PCR data analysis was performed using the comparative threshold cycle (Ct) method by iCycler thermal cycler analyzer program (Bio-Rad, Hercules, CA, USA).

### Database preparation, searching and molecular docking modeling

All modeling calculations were conducted on a four-processor MIPS R16000 Silicon Graphics Tezro running Sybyl 7.1 modeling suite. The structural databases containing over 600,000 structures that consist of available compounds from nearly 50 commercial vendors of chemical databases [20]. Compound libraries were received in the SDF file format [21] and a two dimensional similarity search was run on each database utilizing the DB search command. The resultant hit lists were combined together into a common hit list of three dimensional (3D) SLNs. The DBslnfilter was used to filter out structures containing the following properties: mixtures, metals, isotopes, no 3D coordinates, MW<100, or MW>500. The hit list manager was applied to eliminate all compounds except those containing carboxylates, phosphates and sulfonamides. KIX domain coordinates were obtained by averaging the NMR structures of KIX domain (PDB ID: IKDX). KIX and NASTRp coordinate was generated using phenix.elbow [22]. Docking calculations were performed by using HEX 6.3 [23]. The correlation type used in docking is "Shape+Electrostatistics". The compound similarity screening was performed by using PubChem Compounds.

### Flow cytometric analysis and apoptosis assay

Flow cytometry and TUNEL assays were performed as described previously [12]. NCI-H441 human lung adenocarcinoma cells were treated with NASTRp followed staining with PI/RNase staining buffer (BD Biosciences, San Jose, CA, USA). Cell cycle distribution was analyzed by BD LSRII Flow cytometer (BD Biosciences) and FlowJo software (Treestar, Ashland, OR, USA). For TUNEL assay, A549 human lung adenocarcinoma cells were treated with NASTRp for 48 hours and followed staining with ApopTag Fluorescein Direct In Situ Apoptosis Detection Kit (EMD Millipore, Billerica, MA, USA) according to the manufacturer’s instructions.

### Immunobloting, co-immunoprecipitation and Immunofluorescence assays

Sodium dodecyl sulfate polyacrylamide gel electrophoresis (SDS-PAGE) and western blotting were performed to analyze the expression of various proteins. Cell lysates were lysed by RIPA lysis buffer (10 mM Tris-HCl, pH8.0, 150 mM NaCl, 5 mM EDTA, pH8.0, 0.1% SDS, 1% Sodium deoxycholate, 1% NP-40) supplemented with Complete EDTA-free protease and phosphatase inhibitors cocktails (Roche, South San Francisco, CA, USA) and subjected to immunoblotting using various antibodies. The following antibodies were used: anti-Cyclin A2 (#4656), anti-cyclin B1 (#4138), anti-cyclin E2 (#4132), anti-LC3B (#3868), anti-ATG7 (#8558), anti-ATG5 (#12994), anti-GRP78/Bip (#3177), anti-Caspase 3 (#9665), anti-cleaved Caspase 3 (#9579), anti-Phospho-CREB (S133; #9198) and anti-Bim (#2933) from Cell Signaling Technology, anti-Cyclin D1 from Epitomics (#2261–1, Burlingame, CA, USA), anti-p62/SQSTM1 from America Research Products (03-GP62-C, Waltham, MA, USA), anti-CHOP from Novus Biologicals (NBP2-13172, Littleton, CO, USA), anti-β-actin and anti-FLAG from Sigma-Aldrich (A2228). For co-immunoprecipiation assay, HEK293T cells were co-transfected with pcDNA3-HA-KIX (CBP; amino acids 586–666) and pcDNA3-Myc-KID (CREB; amino acids 87–146 from transcript variant A) using Lipofectamine 2000 (Life Technologies). 48 hours after transfection, cells were pre-treated with NASTRp for 3 hours following 10 μM forskolin (Sigma-Aldrich) administration for additional 1 hour. Cell lysates were prepared with IP buffer (50 mM Tris-HCl, pH7.4, 150 mM NaCl, 1 mM EDTA, pH8.0, 1 mM EGTA, 1 mM DTT, 10% glycerol, 0.5% NP-40, 0.5% Triton X-100) supplemented with Complete EDTA-free protease and phosphatase inhibitors cocktails. Pre-cleaned lysates were subjected to immunoprecipitation using anti-HA mouse monoclonal antibody (MMS-101P, Covance, Princeton, NJ, USA) with conjugated protein A/G PLUS-agarose (sc-2003, Santa Cruz Biotechnology, Dallas, TX, USA). Using Flag-CREB1 stably expressing 293T cells, these stable cells were transfected with pcDNA3-HA-KIX, followed treatments of forskolin or NASTRp as described above. Immune complexes by pull-down with anti-Phospho-CREB antibody were subjected to SDS-PAGE and western blot. For immunofluorescence assay, A549 human lung adenocarcinoma cell lines were treated with the indicated concentrations for 24 hours and followed fixation with 4% paraformaldehyde in PBS for 10 min at room temperature. Fixed cells were permeabilized with 50 mg/ml digitonin in PBS (D141, Sigma-Aldrich) for additional 10 min. Blocked cells with 3% bovine serum albumin (BSA, A9647, Sigma-Aldrich) were probed with primary antibodies and followed conjugation with Alexa Fluor 488 and 568 donkey anti-Rabbit IgG antibodies (Life Technologies), for p62/SQSTM1 and CHOP, respectively. Stained cells were mounted with Prolong Gold Antifade Reagent with DAPI (Life Technologies) and followed observation under fluorescence microscope (Olympus America Co., Center Valley, PA, USA).

### Kaplan-Meier Plotting analysis

To analyze the correlation between patient survival in lung adenocarcinoma and the expressions of autophagy protein, such as ATG7 (Affymetrix ID: 218670_s_at), ATG5 (210639_s_at) or p62/SQSTM1 (201471_s_at), we adopted the databases results from Kaplan-Meier Plotter (http://www.kmplot.com)[24] with the criteria; (1) overall survival (OS), (2) split patients by median, (3) follow up threshold as 9, (4) Histology as adenocarcinoma, (5) Cox regression as uni-variate and (6) array quality control as excluded biased arrays.

### Statistical Analyses

Each experiment was repeated at least three independent times. For generation of graphs and statistical analyses, Graph Pad Prism software (v.6) was used. *P* values between groups were determined by a two-tails unpaired Student *t* tests.

## Results

### Identification of naphthol analogs as CREB-CBP transcription factor/co-activator complex inhibitors

**Fig 1:**
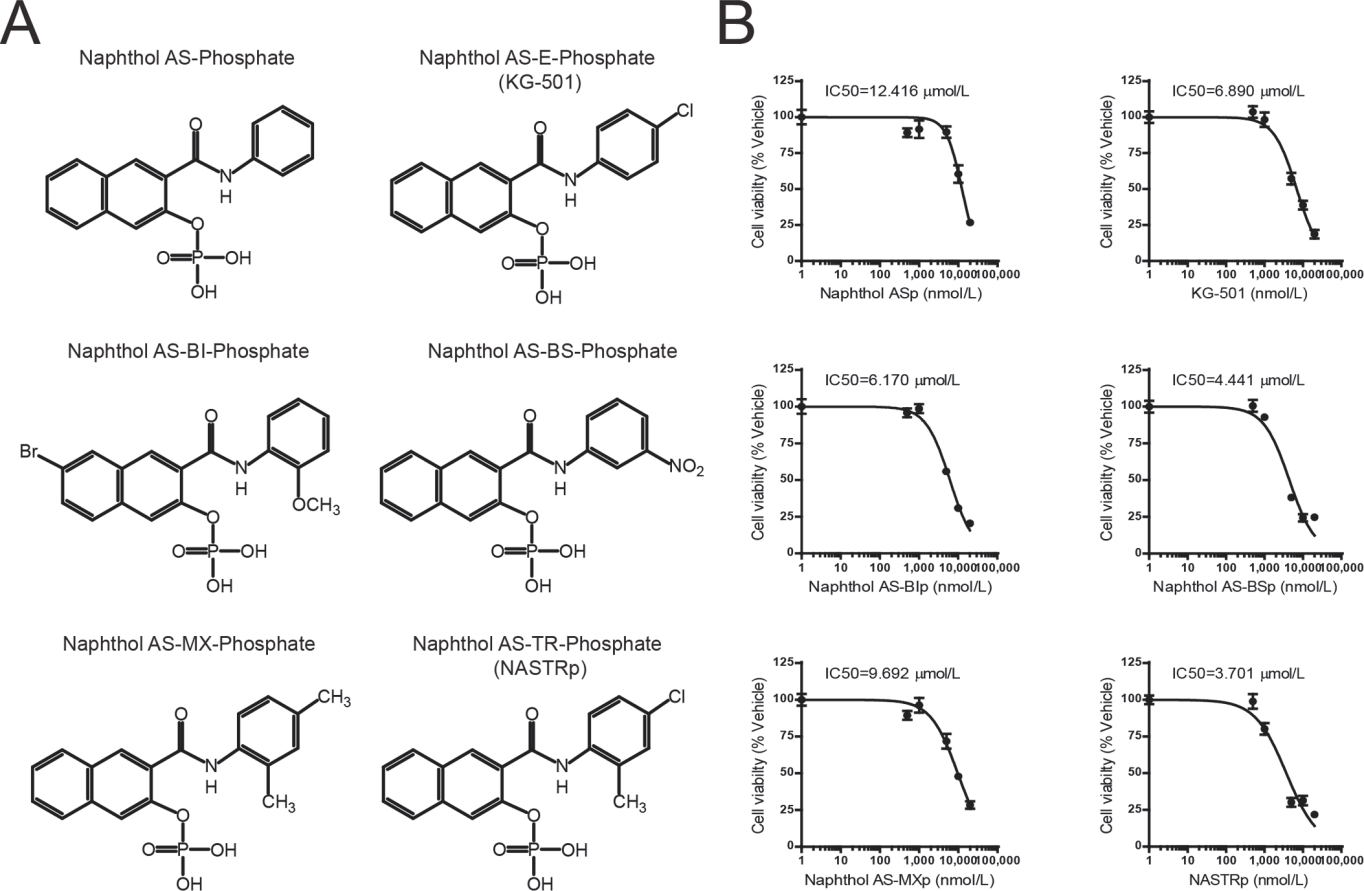
Identification of Naphthol AS-TR phosphate (NASTRp), as an anti-tumor reagent. **(A)** Chemical structures of s_elected naphthol analogs. **(B)** Inhibitory effect of naphthol analogs on cell growth in NCI-H1734 lung adenocarcinoma cell line. Cell viability at 96 hours after treatments was measured by MTT assay with absorbance at 590 nm. The experiments were triplicated and repeated at least three times.

We previously demonstrated that CREB is a critical transcription factor in development of lung cancer through its involvement in cell survival, cell cycle progression, proliferation and apoptosis regulation [5, 6, 10–12]. We first proposed that CREB could be a molecular target for the prevention and treatment of NSCLC [11]. In fact, we demonstrated that CREB regulates IL-1β-regulated CXC chemokines, critical for cell migration and angiogenesis in NSCLC using KG-501 [10]. Thus, these studies lead to the rationale for identification of small molecule inhibitors targeting CREB transcriptional activity described in this study. To identify better compounds, we initially selected a total of 4,547 compounds, which consisted of a two-dimensional similarity cutoff of 80% using the KG-501 structure as a query. Since the phosphate in the KG-501 structure was considered to be an important element, at lease a part, for its solubility, the selection was then further refined to ensure the compounds contained groups considered to be equivalent to the phosphate and this resulted in a 67 candidate compounds. Of these, we identified 6 compounds of naphthol analogs that have anti-cancer effects, as shown Fig 1A.

To determine whether candidate compounds have anti-cancer effect, the human lung adenocarcinoma cell line, NCI-H1734, was exposed to various concentrations of the 6 selected compounds. All compounds showed inhibitory effect on cell proliferation as indicated by IC50s (Fig 1B). Of these, NASTRp was the most potent compound in these proliferation assays as shown: IC50 = 3.701 μmol/L.

We next conducted the docking simulation and calculation using NASTRp and KIX domain of CBP as ligand and receptor, respectively. The result show that NASTRp is close to Arg-600 of the KIX domain of CBP, a critical residue for the CREB-CBP interaction, consistent with KG-501 (S1A Fig; ref. [17]). We further observed that NASTRp completely abolished not only the interaction between KIX and KID in co-transfected HEK293T cells but also the interaction between phosphorylated full-length CREB and KIX in Flag-tagged CREB stably expressing 293T cellsby co-immunoprecipitation assays (S1B and S1C Fig). Therefore, NASTRp was selected and investigated in further biological assays described in this paper.

### Anti-cancer effects of NASTRp in human lung cancer cell lines

**Fig 2:**
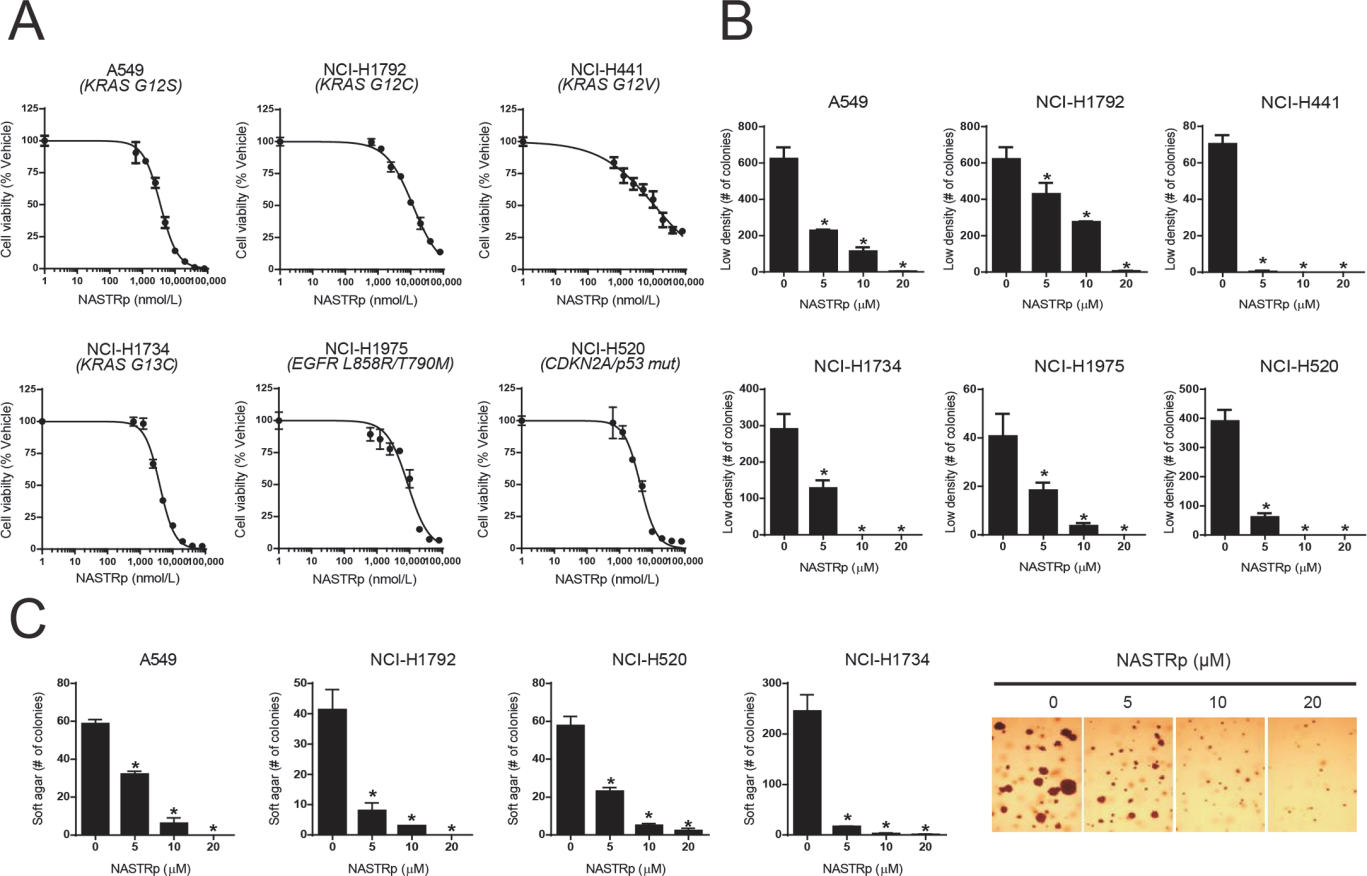
NASTRp inhibits cell proliferation, colony formation and anchorage independent cell growth in human lung cancer cell lines. **(A)** Inhibitory effect of NASTRp on cell proliferation in human lung cancer cell lines. A549, NCI-H1792, NCI-H441, NCI-H1734, NCI-H1975 and NCI-H520 cell lines were treated with various concentrations of NASTRp in RPMI media supplemented with 5% FBS for 96 hours. Cell proliferation was assessed by CellTiter Glo assay as described in METHODS. **(B)** Low density colony formation assay with NASTRp treatment. Cells were plated as single-cell cultures and treated with various concentrations of NASTRp allowed to culture until large colonies were visible. Colonies were stained with crystal violet and photographed to count. **(C)** Suppression of Anchorage independent growth by NASTRp. Cells were plated with low density of agarose (0.4%) containing various concentrations of NASTRp. NCI-H1734 cells were shown as representative images of soft agar assay. Data are presented as the mean ± SD of triplicate wells. *P < 0.05 versus vehicle.

To determine the anti-cancer effiect of NASTRp, several lung cancer cell lines were selected for cell proliferation assay. Initially we chose cells harboring *KRAS* mutations; A549 (KRAS-G12S), NCI-H1792 (KRAS-G12C), NCI-H441 (KRAS-G12V), and NCI-H1734 (KRAS-G13C). To determine the inhibitory effect on cell proliferation, each cell line was exposed to various doses of NASTRp. Consistent with the anti-proliferative effect of NASTRp in NCI-H1734 cells, as shown in Fig 2A, NASTRp dramatically suppressed cell proliferation in all of the lung cancer cell lines with *KRAS* mutations we tested (IC50 = A549, 3.574 μM; NCI-H1792, 11.769 μM; NCI-H441, 11.074 μM; NCI-H1734, 4.025 μM). Given the potent inhibitory effect on cell proliferation of NASTRp, we extended lung cancer cells to lung cancer cell lines harboring *EGFR* mutations, NCI-H1975 (EGFR-L858R/T790M) and a squamous cell carcinoma with highly expressing FGFR and CREB, NCI-H520 (IC50 = NCI-H1975, 8.891 μM; NCI-H520, 4.363 μM; ref. [11]). We also observed that NASTRp significantly inhibited cell growth in low serum contained condition during the treatment (S2 Fig). We found that NASTRp showed significant anti-proliferative effect on theses lung cancer cell lines regardless of its genetic mutation status.

Next, we characterized whether NASTRp affects colony formation and anchorage independent cell growth. Consistent with the inhibitory effect of NASTRp on cell proliferation, colony numbers of all the lung cancer cells were dramatically decreased by NASTRp treatment in anchorage-dependent and independent cell growth (Fig 2B and 2C, S3 Fig). To determine whether the anti-cancer effect of NASTRp is specific to lung cancer, we conducted similar cell proliferation and colony formation assays in pancreatic and breast cancer cells. We observed the inhibitory effects of NASTRp on cell proliferation and colony formation in pancreatic cancer cells (PANC-1, AsPC-1 and SU.86.86.) and breast cancer cells (MCF-7, MDA-MB-231 and SKBR3) as well (S4 Fig). Thus, these results suggest that NASTRp inhibits cell proliferation, colony formation, and anchorage-independent growth in human cancer cells, including at least, lung, pancreatic, and breast cancer cell lines.

### Induction of cell cycle arrest through down-regulation of cyclins by NASTRp

**Fig 3:**
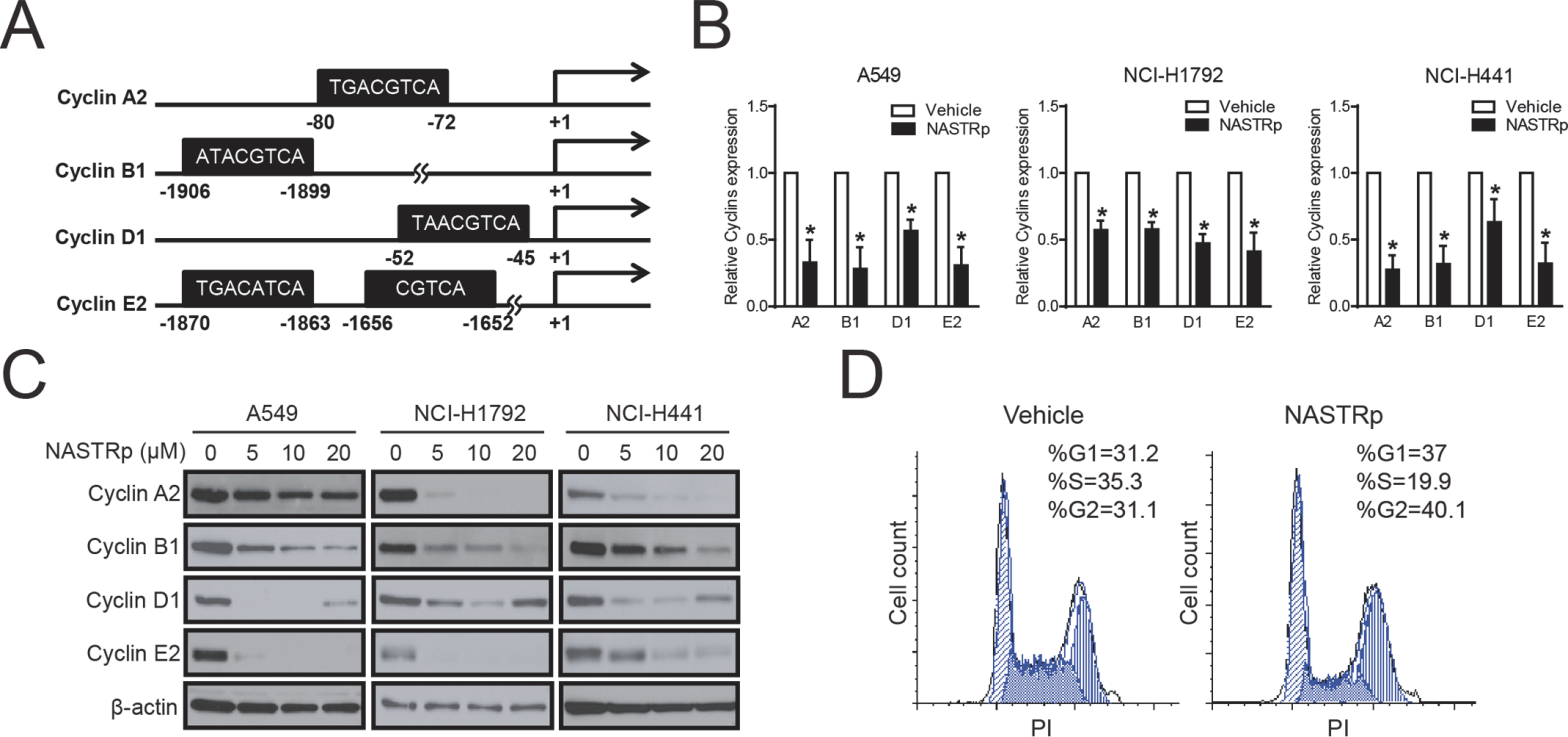
Down-regulation of Cyclins by NASTRp causes cell cycle arrest. **(A)** CRE in the promoters of the cyclin genes. Sequence analysis of the promoters of the genes indicated the potential binding sites for CREB-CBP. **(B and C)** Effect of NASTRp of the expression of cyclins in RNA (**B**, qRT-PCR) and protein (**C**, Western blot) in A549 (left), NCI-H1792 (middle) and NCI-H441 (right). Vehicle control is set to 1 in qRT-PCR assay. For qRT-PCR data are represented as the mean ± SD of three independent experiments performed in triplicate. *P < 0.05 versus vehicle. **(D)** NCI-H441 cells were treated with 10 μM NASTRp for 24 hours and cell cycle distribution was determined by propidium iodide staining followed by FACS analysis.

It has been shown that CREB regulates cell cycle progression through up-regulation of cyclin proteins including cyclin D1 and cyclin A2, which contain CRE elements in their promoter regions [25, 26]. We also confirmed that the cyclins contain CRE elements in their promoters by sequence-based analysis using public databases (e.g. BioBase or TESS). As shown in Fig 3A, cyclin A2, B1, D1, and E2 contain putative CRE regions in their promoters. To determine whether a CREB inhibitor, NASTRp indeed downregulates cyclines, we examined the expression levels of cell cycle-related cyclins under NASTRp treatment by qRT-PCR and western blot assays. As shown Fig 3B and 3C, NASTRp dramatically down-regulated the expressions of cyclins including cyclin A2, B1, D1 and E2 at the mRNA and protein levels. Moreover, NASTRp reduced population of cells in S-phase cell cycle and led to cell cycle arrest at G1 and G2 phases in NCI-H441 cells (Fig 3D). These data indicate that NASTRp suppresses NSCLC cell proliferation, at least in part, through down-regulation of key regulators of cell cycle, cyclins.

### Suppression of autophagy and induction of ER stress and cell death by NASTRp

Interestingly, we observed several vacuole-like structures in the cytosol of NASTRp-treated cells. We then hypothesized that NASTRp might suppress cytoprotective autophagy counteracting a tumor-promoting role to function as an anti-cancer drug. Autophagy is often considered as an alternative source of nutrition for cancer cells survival when they are under limited growth supply. Thus, we examined autophagy markers, such as the LC3B modification and p62/SQSTM1 degradation under NASTRp treatment in human NSCLC cells. Of note, p62 is a key molecule managing autophagic clearance of polyubiquitinated proteins through directly binding to LC3 in autophagosomes [27]. Defect of autophagy causes accumulation of p62, ubiquitin conjugating protein aggregates and damaged organelles particularly mitochondria [28, 29].

**Fig 4:**
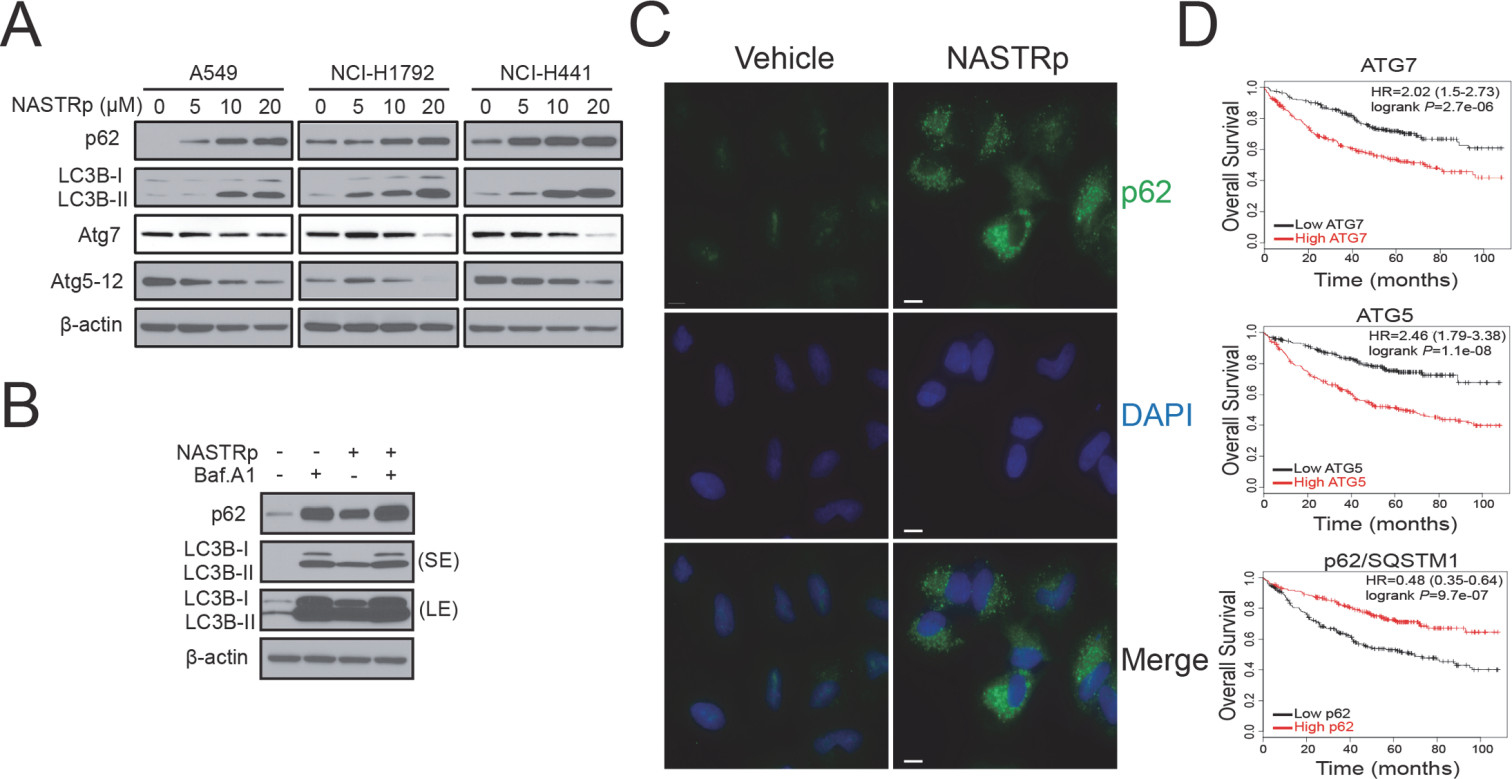
NASTRp suppresses tumor-promoting autophagy in human cancer cells. **(A)** Cells were treated with various concentrations of NASTRp in RPMI-1640 media supplemented with 5% FBS for 24 hours. Cell lysates were subjected to western blot assay with the indicated antibodies. **(B)** Inhibition of autophagy flux by NASTRp. NCI-H441 cells were treated with 20 μM NASTRp in the presence or absence of bafilomycin A1 (100 nM) for 24 hours. Cell lysates were subjected to SDS-PAGE/Western blot analysis using the indicated antibodies. **(C)** Accumulation of p62 aggregates by NASTRp. A549 cells were treated with 20 μM NASTRp for 24 hours. Treated cells were followed the immunofluorescence staining with anti-p62 antibody. Scale bar: 10 μm. **(D)** Potential clinical benefit of NASTRp in lung adenocarcinoma patients. Kaplan-Meier survival curves for human lung adenocarcinoma datasets, which compare the overall survival between tumors with high levels (red) or low levels (black) of ATG7 (left panel), ATG5 (middle panel) and p62/SQSTM1 (right panel) gene expression from Kaplan-Meier Plotter tool. Log-rank *P* values are shown.

We found that in all cell lines examined, p62 dramatically accumulated as a consequence of NASTRp treatment in a dose-dependent manner (Fig 4A). While other critical autophagy related proteins including ATG7 and ATG5-12 conjugation levels were significantly decreased following NASTRp treatment, indicating a suppressive effect of NASTRp on autophagy (Fig 4A). Furthermore, NASTRp blocked the basal autophagy flux as shown by no change of LC3B modification or p62 degradation in the presence of bafilomycin A1 (a V-ATPase inhibitor), which blocks the late steps of autophagy (Fig 4B).

The accumulation of p62 was further confirmed cells using immunofluorescence in A549. Consistent with the immunobloting result, p62 aggregates (>2–4 μm) significantly increased in the cytosol following NASTRp treatment compared to vehicle (Fig 4C). These results are in line with the hypothesis that NASTRp can suppress the tumor-promoting/cytoprotective autophagy, at least in part, through ATG7 down-regulation and blocking autophagy flux as shown by accumulation of LC3B-II and p62.

Since the correlation between patient survival in lung adenocarcinoma and the expressions of autophagy proteins, such as ATG7, ATG5 or p62 have not yet been reported, we next conducted a survival analysis of lung adenocarcinoma patients scored for *ATG7*, *ATG5* or *p62* mRNA expressions using public databases. In the *ATG7*-related database (ID: 218673_s_at), there are 242 (49.8%) cases for high *ATG7* expression and 244 (50.2%) cases for low *ATG7* expression. The Kaplan-Meier survival analysis shows that patients harboring low *ATG7* expression had a significantly improved survival compared with the ones with high *ATG7* expression (Fig 4D, top, *P* = 2.7e-06). The patients with low *ATG5* expression had better survival consistent with the *ATG7* cases (Fig 4D, middle, *P* = 1.1e-08). In contrast, the patients with high expression of *p62* had significantly better survival outcome compared with the patients with low *p62* expression (Fig 4D, bottom, *P* = 9.7e-07).

**Fig 5:**
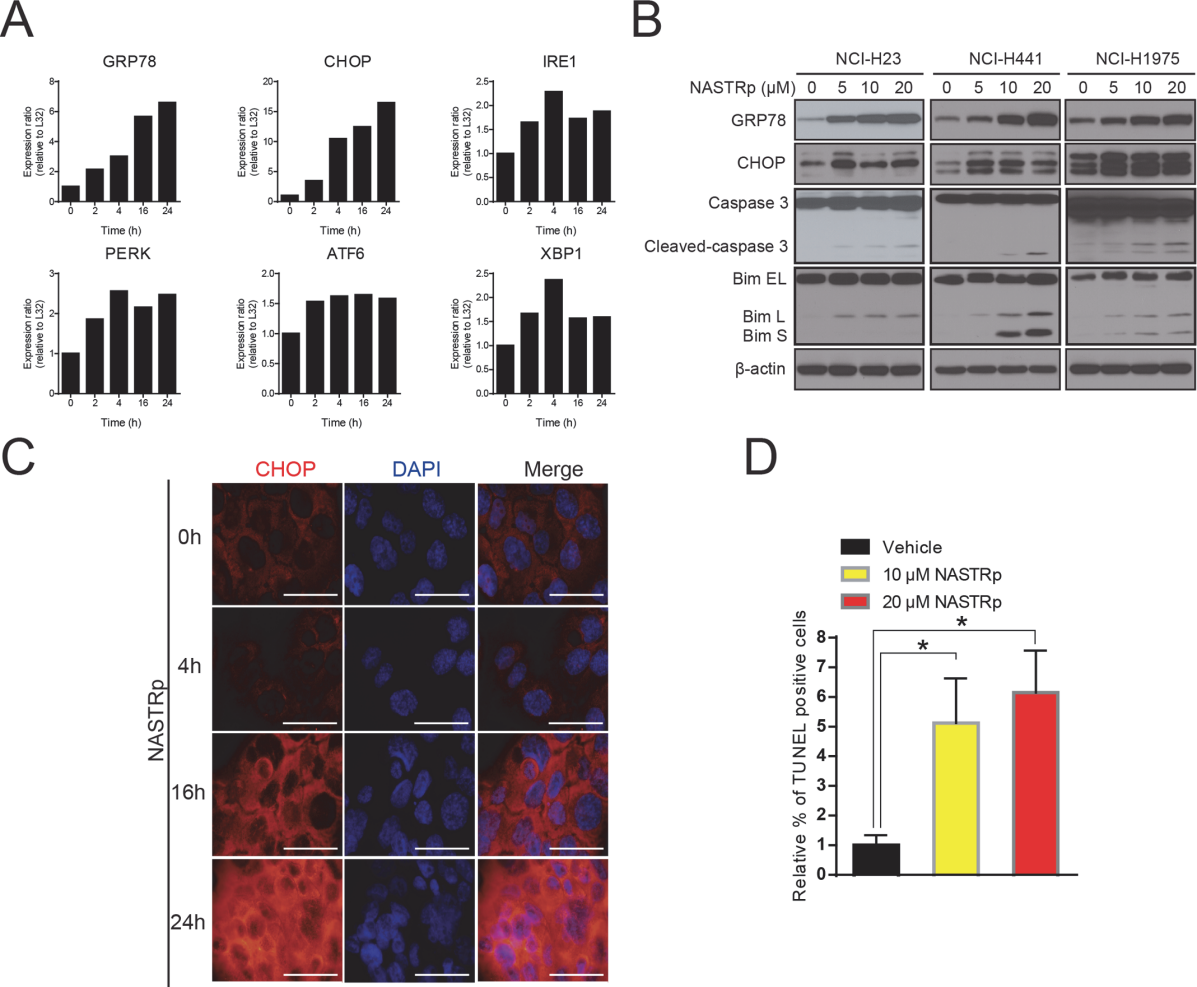
NASTRp induces ER stress and eventually leads to cell death with Bim up-regulation. **(A)** qRT-PCR analysis of biomarkers of ER stress/UPR related genes in NCI-H441 treated with 20 μM NASTRp in time course manner. **(B)** NASTRp induced ER stress and activated UPR led to apoptosis with Bim induction in NSCLC cell lines. Cells were treated with the indicated concentrations of NASTRp for 24 hours and cell lysates were subjected to SDS-PAGE/Western blot analysis using the indicated antibodies. **(C)** Upregulation of CHOP by NASTRp. NCI-H441 cells were treated with 20 NASTRp for 0–24 hours. At the indicated time points, cells were fixed and subjected to immunofluorescence assay with anti-CHOP antibody. Cell images were microphotographed using fluorescence microscopy at 60X magnification. Scale bars: white; 10 μm. **(D)** TUNEL-positive cells in NASTRp-treated (for 48 hours) A549 cells were counted and analyzed as relative % of TUNEL-positive cells. *P < 0.05 versus vehicle.

It has been reported that defective autophagy through down-regulated ATG7 resulted in elevated ER stress in metabolic liver disease [30]. Given the suppression of ATG7 by NASTRp, we further examined expression of ER stress markers, such as GRP78, CHOP, IRE1, PERK, ATF6 and XBP1, to determine whether NASTRp induces ER stress in lung cancer cells. As shown in Fig 5A, NASTRp induced ER stress and activated unfolded protein response (UPR), demonstrated by the up-regulation of GRP78 [31] and CHOP. In addition, the expression levels of other genes related to ER stress, such as IRE1, ATF6, PERK and XBP1 also increased following NASTRp treatment in human cancer cells (Fig 5A). We confirmed that NASTRp up-regulated the level of GRP78 and CHOP proteins in a time and dose-dependent manners (Fig 5A and 5B) and observed a dramatic increase of CHOP expression in the nucleus (Fig 5C). The ER stress became insurmountable in the presence of NASTRp and led to cell death as shown by increased cleaved caspase-3, Bim expression and TUNEL-positive dots (Fig 5B and 5D).

**Fig 6:**
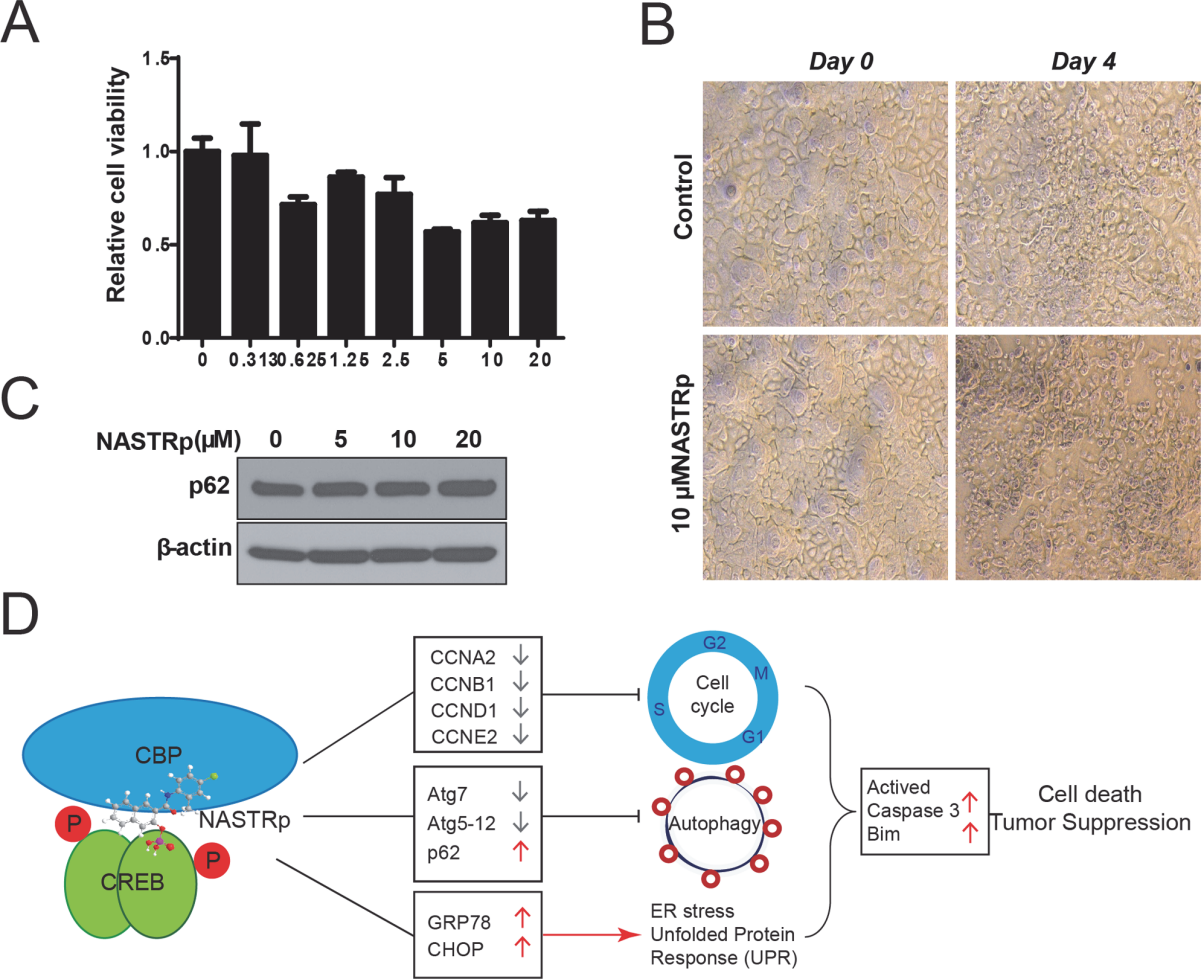
NASTRp targets cancer cell rather than normal epithelial cells. **(A)** Cell viability assay in response to NASTRp of NHTBE cells. **(B)** NHTBE cells which were cultured in 3D organotypic air-liquid interface (ALI) system under NASTRp treatment. NHTBE cells were treated with 10 μM NASTRp for 96 hours. Representative pictures were obtained using phase-contrast light microscopy. **(C)** Unchanged p62 level in NASTRp-treated NHTBE cell. NHTBE cells were treated with the indicated doses of NASTRp for 24 hours. Cell lysates were subjected to western blotting using anti-p62 antibody. **(D)** Schematic model of anti-cancer effects of NASTRp. NASTRp targeting the transcription factor complex, CREB-CBP leads to cell cycle arrest by down-regulating CREB target genes, including cell cycle related genes. Further NASTRp suppresses tumor-promoting autophagy concomitant with induction of ER stress. NASTRp finally triggers cell death in the lung tumor.

We then evaluated the on-target effect of NASTRp using normal human tracheobronchial epithelial (NHTBE) cells in two-dimensional (regular tissue culture condition) and three-dimentsional organotypic (air-liquid interface culture condition; ALI) culture systems [5, 17–19]. Although there was minor killing effect of NASTRp on NHTBE cells, cell viability was not decreased lower than 50%, even 20 μM of NASTRp treatment (Fig 6A and 6B). Whereas p62 was accumulated in lung cancer cells, p62 in NHTBE cells was unchangeable following NASTRp treatment (Fig 6C). These data indicate that NASTRp may specifically target into cancer cell rather than normal cell, especially within therapeutic window ranges. Taken together, these results suggest that NASTRp suppresses the tumor-promoting role of autophagy through ATG7 down-regulation and p62 accumulation, induces ER stress with activation of UPR, and eventually leads to cell death in human NSCLC cells (Fig 6D).

## Discussion

Since CREB is constitutively overexpressed and hyperphosphorylated in human lung cancer tissues and cells and considered as a critical transcription factor in cancer pathogenesis [9–12], we identified several small molecule inhibitors of the CREB-CBP transcription factor/co-activator that target the binding domain KIX of CBP and prevent the formation of a complex with the KID domain of CREB. We further demonstrated the effect of these inhibitors, but in particular, NASTRp in lung cancer cell lines. Our study highlights the anti-tumor effect of the transcription factors CREB-CBP inhibitor, NASTRp, in *in vitro*, through regulation of cell cycle, tumor-promoting autophagy, ER stress and cell death (Fig 6D). Moreover, these effects of NASTRp may specifically target cancer cell, rather than normal cell, in particular, therapeutic window. We provide evidence that the CREB-CBP complex may be a potential therapeutic target in human cancers and its inhibitors may be an alternate treatment option for lung cancer patients and potentially other tumor types, including pancreatic and breast cancers.

NASTRp is modified from naphthol analog, KG-501, which was originally identified by Montminy group [16]. Consistent with KG-501 docking model, NASTRp showed the localization to Arg-600 which is a critical residue for CREB-CBP interaction. Moreover, NASTRp completely abolished the association between KIX(CBP) and KID in the cells. Thus, we expect that NASTRp can inhibit CREB transcriptional activity with disruption of the CREB-CBP interaction through integrating into the grooves adjacent to Arg-600 the KIX domain of CBP, as shown S1A and S1B Fig.

CBP and its homolog p300 are transcription co-activators possessing multiple domains and histone acetyl transferase activity that collectively mediate transcriptional activity of over 100 transcription factors involved in cell growth and development. Through their interactions to transcription factors, CBP and p300 induce or stabilize the transcription factors to sustain their activities as oncogenic factors. In this regard, NASTRp can not only regulate the transcriptional regulation of CREB, but also other CBP/p300-interacting transcription factors and as such act as a broad anti-cancer agent, by regulating processes such as cell cycle progression, autophagy, ER stress and cell death.

Autophagy is a highly orchestrated intracellular degradation process that is not only regulated at basal levels in most tissues as routine turnover of cytoplasmic components, but also by various environmental stresses [32]. Thus, recent studies have pointed to dysfunction of autophagy as a novel hallmark of cancer that is central to the pathogenesis of human malignancy [33]. Context dependent, autophagy can both suppress cancer initiation and provide a survival advantage for established cancers. Many cancer cells with mutant *RAS* are highly dependent on autophagy for survival under basal, and especially stress conditions [34]. It is interesting that NASTRp also regulates tumor-promoting role of autophagy which supports cancer cell survival under limited growth supply in tumor mass. Emerging evidences including our finding suggest that blocking of autophagy may be a valuable therapeutic approach for autophagy-addicted aggressive cancers [34, 35]. NASTRp suppresses not only ATG7 and ATG5-12 levels, but also blocks autophagic flux as shown by LC3B-II and p62 accumulation (Fig 4A–4C). Recent studies showed that deficiency of *Atg7* in *Kras*-driven lung cancer mouse models display suppressed lung tumor growth and prolonged survival, consistent with our finding [35]. Thus, we predicted that blockage of autophagy may be critical for therapeutic efficacy and data correlating ATG7 expression with survival support this (Fig 4D). Our results suggest that NASTRp may be a novel cancer therapy by inhibiting cell cycle as well as autophagy that will provide benefits for lung cancer treatment in advanced and/or KRAS mutant lung cancers. Future work will be required to address the molecular mechanism by which NASTRp suppresses autophagy or related molecules such as ATG7, ATG5 and p62 in lung cancers.

In particular, cancer cells frequently display elevated ER stress and defective autophagy leads to cells being vulnerable to ER stress, further insurmountable ER stress causes cell death [30, 36]. Interestingly, we found that NASTRp also induces up-regulations of ER stress-responded proteins, such as GRP78/Bip and CHOP/GADD153 concomitant with suppression of autophagy, further consequence NASTRp causes cell death with Bim induction (Fig 5A–5C). Although the molecular mechanism has not been yet elucidated, it is noteworthy that transcriptional regulation of CREB plays a functional role in mediating the multiple effects of NASTRp. However, the possibility remains that other, yet unknown and CREB-independent mechanisms may be involved in CBP/p300’s role in tumorigenesis. Future studies are warranted to fully understand the anti-tumor effect of NASTRp in lung cancers.

Although the high IC50 of NASTRp would be concerned as off-target or non-specific effects, we observed that NASTRp without phosphate group (NASTR) showed ~3 fold more potent anti-tumorigenic effects as shown much lower IC50 (~1–7 μM), but the solubility was poor (data not shown). Thus, we speculate that high IC50 of NASTRp might be due to phosphate group in the structure which is required to improvement of solubility; this is considered to be important, especially for *in vivo* study. It will be worthwhile to test the anti-cancer efficacy of NASTRp in *in vivo* mouse model, along with the improvement of solubility as well as potency and delivery methods in the future.

We conclude that a small-molecule inhibitor, NASTRp targeting the CREB-CBP transcription complex is a novel, potent anti-cancer agent showing multiple effects on critical pathways in cancer cell biology. Our data have suggested a new class of targeted therapies in cancer, which extend activity to the KRAS and p53 mutants segment that is presently not well served. These observations would also suggest as a potential therapeutic strategy for the patients who harbor overexpressed CREB and/or other oncogenic mutations that activate CREB in human cancers, especially lung cancer.

## Supporting Information

S1 Fig NASTRp binds to KIX domain of CBP to interrupt the interaction between CREB and CBP. **(A)** Docking calculation of NASTRp into KIX domain of CBP. Five out of ten top docking calculations show NASTRp close to a binding pocket near Arg-600 of KIX that consists with NMR observations. The figures were generated by Chimera 1.8 [37]. **(B)** Disruption of KIX-KID complex by NASTRp. HEK293T cells were co-transfected with HA-KIX and Myc-KID. 48 hours after transfection, cells were pre-treated with 20 or 40 μM NASTRp for 3 hours, followed addition of 10 μM forskolin for additional 1 hour. Cell lysates were subjected to immunoprecipitation assay and western blotting. **(C)** Flag-CREB stably expressing 293T cells were transfected with HA-KIX, followed treatment with NASTRp and/or forskolin. Cell lysates were subjected to immunoprecipitation assay using anti-Phospho-CREB (S133) antibody.
(TIF)

S2 Fig The inhibitory effect of cell growth by NASTRp under low serum condition in human lung cancer cell lines. Cells were seeded and followed incubation with medium supplemented with 2% FBS for 2, 4 and 6 days. At the indicated times, cells were stained with crystal violet and then extracted with 10% acetic acid followed measurement of absorbance at 490 nm. Experiment were triplicated and independently repeated three times. The Data present mean ± SD for triplicate in three independent experiments. *P<0.05.
(TIF)

S3 Fig The inhibitory effect of colony formation by NASTRp in human cancer cell lines. Representative pictures of each group were shown. Experiment were triplicated and independently repeated three times.
(TIF)

S4 Fig NASTRp also suppresses cell proliferation and colony formation of pancreatic and breast cancer cells. (A) Pancreatic cancer cells (SU.86.86, PANC-1, AsPC-1) and (B) breast cancer cells (SKBR3, MDA-MB-231, MCF-7) were plated into 96-well plates at 2000 cells/well in 100 μl medium supplemented with 10% FBS and treated with 0–80 μM of NASTR for 96 hours. The treated cells were assayed for cell proliferation using CellTiter-Glo cell viability kit (Promega). The Data present mean ± SD for triplicate in three independent experiments. (C) Representative images of colony formation assay in the absence or presence of NASTR in pancreatic and breast cancer cells.
(TIF)

## References

[1] Siegel R, Naishadham D, Jemal A. Cancer statistics, 2013. CA Cancer J Clin. 2013;63(1):11–30. 10.3322/caac.21166 23335087

[2] Downward J. Targeting RAS signalling pathways in cancer therapy. Nat Rev Cancer. 2003;3(1):11–22. 12509763 10.1038/nrc969

[3] Pylayeva-Gupta Y, Grabocka E, Bar-Sagi D. RAS oncogenes: weaving a tumorigenic web. Nat Rev Cancer. 2011;11(11):761–74. 10.1038/nrc3106 21993244 PMC3632399

[4] Cheng JC, Kinjo K, Judelson DR, Chang J, Wu WS, Schmid I, et al CREB is a critical regulator of normal hematopoiesis and leukemogenesis. Blood. 2008;111(3):1182–92. 17975014 10.1182/blood-2007-04-083600 PMC2214769

[5] Aggarwal S, Kim SW, Cheon K, Tabassam FH, Yoon JH, Koo JS. Nonclassical action of retinoic acid on the activation of the cAMP response element-binding protein in normal human bronchial epithelial cells. Mol Biol Cell. 2006;17(2):566–75. 16280361 10.1091/mbc.E05-06-0519 PMC1356569

[6] Kim SW, Hong JS, Ryu SH, Chung WC, Yoon JH, Koo JS. Regulation of mucin gene expression by CREB via a nonclassical retinoic acid signaling pathway. Molecular and cellular biology. 2007;27(19):6933–47. 17646388 10.1128/MCB.02385-06 PMC2099243

[7] Liu Y, Dentin R, Chen D, Hedrick S, Ravnskjaer K, Schenk S, et al A fasting inducible switch modulates gluconeogenesis via activator/coactivator exchange. Nature. 2008;456(7219):269–73. 10.1038/nature07349 18849969 PMC2597669

[8] Yin JC, Del Vecchio M, Zhou H, Tully T. CREB as a memory modulator: induced expression of a dCREB2 activator isoform enhances long-term memory in Drosophila. Cell. 1995;81(1):107–15. 7720066 10.1016/0092-8674(95)90375-5

[9] Conkright MD, Montminy M. CREB: the unindicted cancer co-conspirator. Trends Cell Biol. 2005;15(9):457–9. 16084096 10.1016/j.tcb.2005.07.007

[10] Sun H, Chung WC, Ryu SH, Ju Z, Tran HT, Kim E, et al Cyclic AMP-responsive element binding protein- and nuclear factor-kappaB-regulated CXC chemokine gene expression in lung carcinogenesis. Cancer Prev Res (Phila). 2008;1(5):316–28. 10.1158/1940-6207.CAPR-07-0002 19138976 PMC2768131

[11] Seo HS, Liu DD, Bekele BN, Kim MK, Pisters K, Lippman SM, et al Cyclic AMP response element-binding protein overexpression: a feature associated with negative prognosis in never smokers with non-small cell lung cancer. Cancer Res. 2008;68(15):6065–73. 10.1158/0008-5472.CAN-07-5376 18676828 PMC3058903

[12] Aggarwal S, Kim SW, Ryu SH, Chung WC, Koo JS. Growth suppression of lung cancer cells by targeting cyclic AMP response element-binding protein. Cancer Res. 2008;68(4):981–8. 10.1158/0008-5472.CAN-06-0249 18281471 PMC2921320

[13] Moreno CS, Beresford GW, Louis-Plence P, Morris AC, Boss JM. CREB regulates MHC class II expression in a CIITA-dependent manner. Immunity. 1999;10(2):143–51. 10072067 10.1016/s1074-7613(00)80015-1

[14] Parker D, Ferreri K, Nakajima T, LaMorte VJ, Evans R, Koerber SC, et al Phosphorylation of CREB at Ser-133 induces complex formation with CREB-binding protein via a direct mechanism. Mol Cell Biol. 1996;16(2):694–703. 8552098 10.1128/mcb.16.2.694 PMC231049

[15] Kasper LH, Lerach S, Wang J, Wu S, Jeevan T, Brindle PK. CBP/p300 double null cells reveal effect of coactivator level and diversity on CREB transactivation. The EMBO journal. 2010;29(21):3660–72. 10.1038/emboj.2010.235 20859256 PMC2982758

[16] Best JL, Amezcua CA, Mayr B, Flechner L, Murawsky CM, Emerson B, et al Identification of small-molecule antagonists that inhibit an activator: coactivator interaction. Proc Natl Acad Sci U S A. 2004;101(51):17622–7. 15585582 10.1073/pnas.0406374101 PMC539725

[17] Koo JS, Jetten AM, Belloni P, Yoon JH, Kim YD, Nettesheim P. Role of retinoid receptors in the regulation of mucin gene expression by retinoic acid in human tracheobronchial epithelial cells. Biochem J. 1999;338 (Pt 2):351–7. 10024510 PMC1220060

[18] Kim SW, Cheon K, Kim CH, Yoon JH, Hawke DH, Kobayashi R, et al Proteomics-based identification of proteins secreted in apical surface fluid of squamous metaplastic human tracheobronchial epithelial cells cultured by three-dimensional organotypic air-liquid interface method. Cancer Res. 2007;67(14):6565–73. 17638865 10.1158/0008-5472.CAN-06-2783 PMC2958044

[19] Lee J, Ryu SH, Kang SM, Chung WC, Gold KA, Kim ES, et al Prevention of bronchial hyperplasia by EGFR pathway inhibitors in an organotypic culture model. Cancer Prev Res (Phila). 2011;4(8):1306–15. 10.1158/1940-6207.CAPR-10-0364 21505178 PMC3151315

[20] Baurin N, Baker R, Richardson C, Chen I, Foloppe N, Potter A, et al Drug-like annotation and duplicate analysis of a 23-supplier chemical database totalling 2.7 million compounds. J Chem Inf Comput Sci. 2004;44(2):643–51. 15032546 10.1021/ci034260m

[21] Dalby A, Hounshell WD, Gushurst AK, Grier DL. Description of several chemical structure file formats used by computer programs developed at Molecular Design Limited. J Chem Inf Comput Sci. 1992;32(3):244–54.

[22] Moriarty NW, Grosse-Kunstleve RW, Adams PD. electronic Ligand Builder and Optimization Workbench (eLBOW): a tool for ligand coordinate and restraint generation. Acta Crystallogr D Biol Crystallogr. 2009;65(Pt 10):1074–80. 10.1107/S0907444909029436 19770504 PMC2748967

[23] Ritchie DW, Venkatraman V. Ultra-fast FFT protein docking on graphics processors. Bioinformatics. 2010;26(19):2398–405. 10.1093/bioinformatics/btq444 20685958

[24] Gyorffy B, Surowiak P, Budczies J, Lanczky A. Online survival analysis software to assess the prognostic value of biomarkers using transcriptomic data in non-small-cell lung cancer. PLoS One. 2013;8(12):e82241 10.1371/journal.pone.0082241 24367507 PMC3867325

[25] Altarejos JY, Montminy M. CREB and the CRTC co-activators: sensors for hormonal and metabolic signals. Nat Rev Mol Cell Biol. 2011;12(3):141–51. 10.1038/nrm3072 21346730 PMC4324555

[26] Beier F, Lee RJ, Taylor AC, Pestell RG, LuValle P. Identification of the cyclin D1 gene as a target of activating transcription factor 2 in chondrocytes. Proc Natl Acad Sci U S A. 1999;96(4):1433–8. 9990041 10.1073/pnas.96.4.1433 PMC15480

[27] Pankiv S, Clausen TH, Lamark T, Brech A, Bruun JA, Outzen H, et al p62/SQSTM1 binds directly to Atg8/LC3 to facilitate degradation of ubiquitinated protein aggregates by autophagy. J Biol Chem. 2007;282(33):24131–45. 17580304 10.1074/jbc.M702824200

[28] Hara T, Nakamura K, Matsui M, Yamamoto A, Nakahara Y, Suzuki-Migishima R, et al Suppression of basal autophagy in neural cells causes neurodegenerative disease in mice. Nature. 2006;441(7095):885–9. 16625204 10.1038/nature04724

[29] Komatsu M, Waguri S, Koike M, Sou YS, Ueno T, Hara T, et al Homeostatic levels of p62 control cytoplasmic inclusion body formation in autophagy-deficient mice. Cell. 2007;131(6):1149–63. 18083104 10.1016/j.cell.2007.10.035

[30] Yang L, Li P, Fu S, Calay ES, Hotamisligil GS. Defective hepatic autophagy in obesity promotes ER stress and causes insulin resistance. Cell metabolism. 2010;11(6):467–78. 10.1016/j.cmet.2010.04.005 20519119 PMC2881480

[31] Baumeister P, Luo S, Skarnes WC, Sui G, Seto E, Shi Y, et al Endoplasmic reticulum stress induction of the Grp78/BiP promoter: activating mechanisms mediated by YY1 and its interactive chromatin modifiers. Mol Cell Biol. 2005;25(11):4529–40. 15899857 10.1128/MCB.25.11.4529-4540.2005 PMC1140640

[32] Shintani T, Klionsky DJ. Autophagy in health and disease: a double-edged sword. Science. 2004;306(5698):990–5. 15528435 10.1126/science.1099993 PMC1705980

[33] Hanahan D, Weinberg RA. Hallmarks of cancer: the next generation. Cell. 2011;144(5):646–74. 10.1016/j.cell.2011.02.013 21376230

[34] Guo JY, Chen HY, Mathew R, Fan J, Strohecker AM, Karsli-Uzunbas G, et al Activated Ras requires autophagy to maintain oxidative metabolism and tumorigenesis. Genes Dev. 2011;25(5):460–70. 10.1101/gad.2016311 21317241 PMC3049287

[35] Guo JY, Karsli-Uzunbas G, Mathew R, Aisner SC, Kamphorst JJ, Strohecker AM, et al Autophagy suppresses progression of K-ras-induced lung tumors to oncocytomas and maintains lipid homeostasis. Genes Dev. 2013;27(13):1447–61. 10.1101/gad.219642.113 23824538 PMC3713426

[36] Ogata M, Hino S, Saito A, Morikawa K, Kondo S, Kanemoto S, et al Autophagy is activated for cell survival after endoplasmic reticulum stress. Mol Cell Biol. 2006;26(24):9220–31. 17030611 10.1128/MCB.01453-06 PMC1698520

[37] Pettersen EF, Goddard TD, Huang CC, Couch GS, Greenblatt DM, Meng EC, et al UCSF Chimera—a visualization system for exploratory research and analysis. J Comput Chem. 2004;25(13):1605–12. 15264254 10.1002/jcc.20084

