# Supplementary figures and images for: A Novel Small-Molecule Inhibitor Targeting CREB-CBP Complex Possesses Anti-Cancer Effects along with Cell Cycle Regulation, Autophagy Suppression and Endoplasmic Reticulum Stress
Source: PLoS One. 2015 Apr 21;10(4):e0122628. doi: 10.1371/journal.pone.0122628 (PMC4405579; doi:10.1371/journal.pone.0122628)

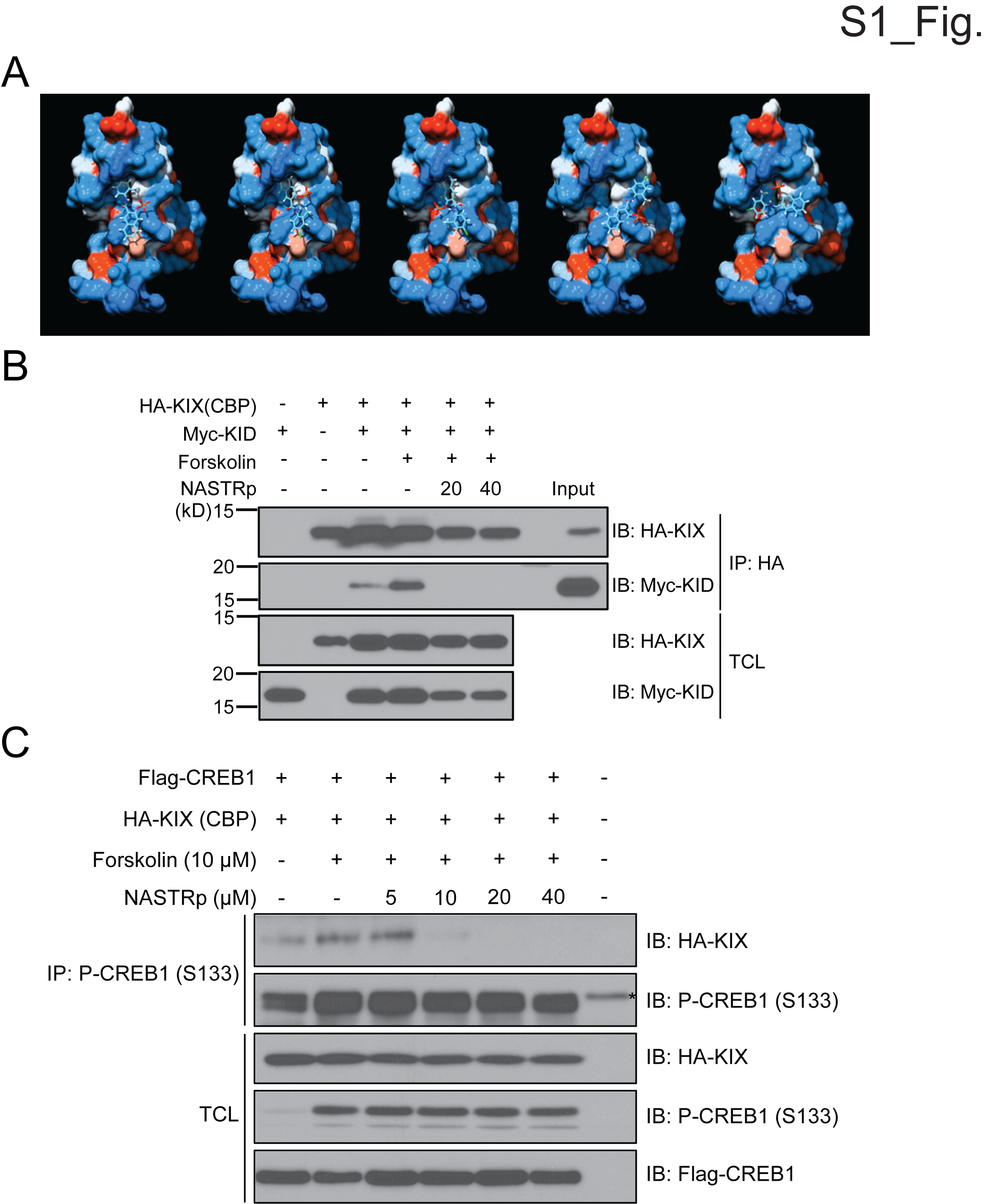

Supplement: S1 Fig — (A) Docking calculation of NASTRp into KIX domain of CBP. Five out of ten top docking calculations show NASTRp close to a binding pocket near Arg-600 of KIX that consists with NMR observations. The figures were generated by Chimera 1.8 [37]. (B) Disruption of KIX-KID complex by NASTRp. HEK293T cells were co-transfected with HA-KIX and Myc-KID. 48 hours after transfection, cells were pre-treated with 20 or 40 μM NASTRp for 3 hours, followed addition of 10 μM forskolin for additional 1 hour. Cell lysates were subjected to immunoprecipitation assay and western blotting. (C) Flag-CREB stably expressing 293T cells were transfected with HA-KIX, followed treatment with NASTRp and/or forskolin. Cell lysates were subjected to immunoprecipitation assay using anti-Phospho-CREB (S133) antibody. (TIF) [file pone.0122628.s001.tif]

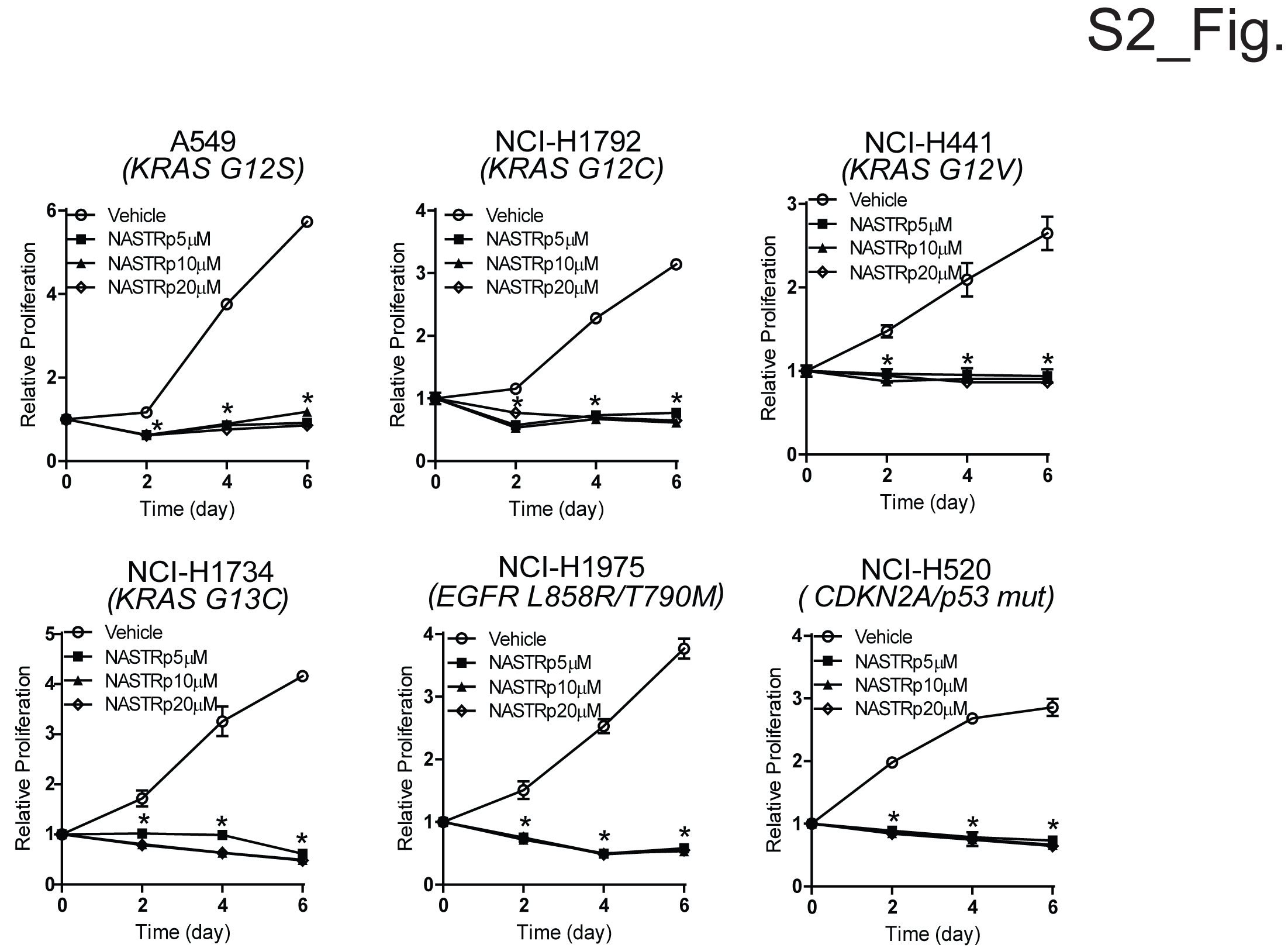

Supplement: S2 Fig — Cells were seeded and followed incubation with medium supplemented with 2% FBS for 2, 4 and 6 days. At the indicated times, cells were stained with crystal violet and then extracted with 10% acetic acid followed measurement of absorbance at 490 nm. Experiment were triplicated and independently repeated three times. The Data present mean ± SD for triplicate in three independent experiments. *P<0.05. (TIF) [file pone.0122628.s002.tif]

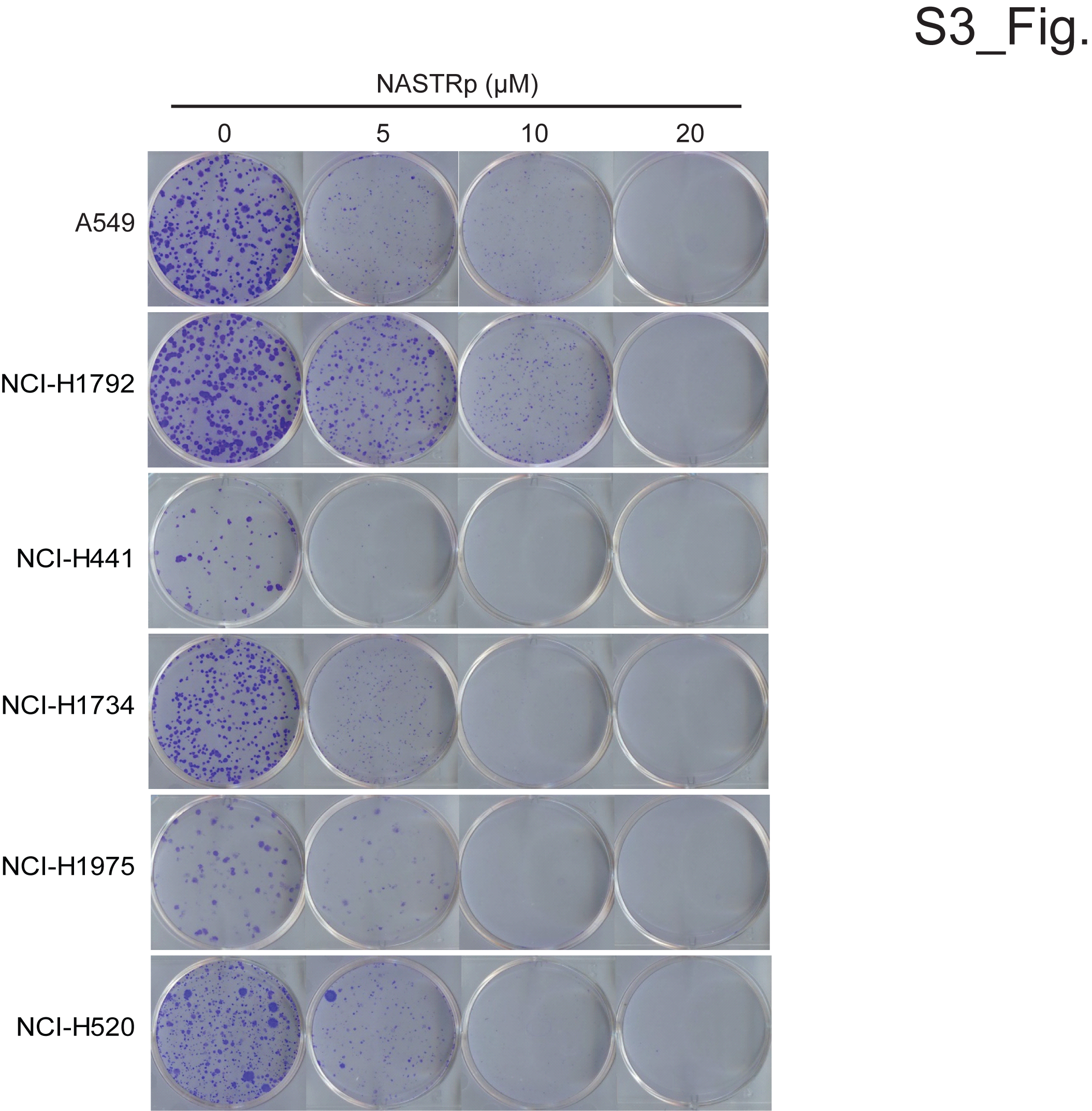

Supplement: S3 Fig — Representative pictures of each group were shown. Experiment were triplicated and independently repeated three times. (TIF) [file pone.0122628.s003.tif]

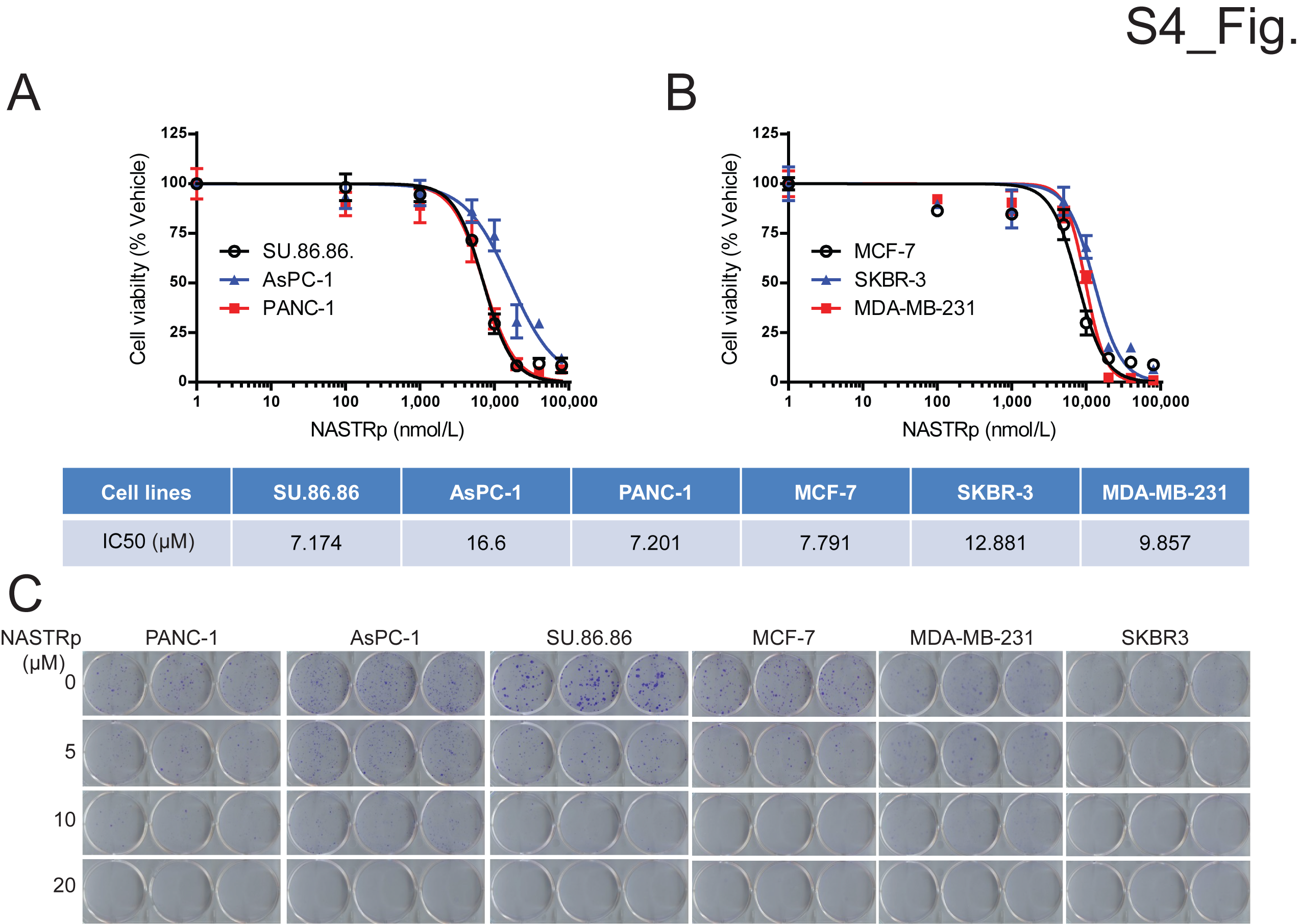

Supplement: S4 Fig — (A) Pancreatic cancer cells (SU.86.86, PANC-1, AsPC-1) and (B) breast cancer cells (SKBR3, MDA-MB-231, MCF-7) were plated into 96-well plates at 2000 cells/well in 100 μl medium supplemented with 10% FBS and treated with 0–80 μM of NASTR for 96 hours. The treated cells were assayed for cell proliferation using CellTiter-Glo cell viability kit (Promega). The Data present mean ± SD for triplicate in three independent experiments. (C) Representative images of colony formation assay in the absence or presence of NASTR in pancreatic and breast cancer cells. (TIF) [file pone.0122628.s004.tif]
